# HDR-based CRISPR/Cas9-mediated Knockout of PD-L1 in C57BL/6 Mice

**DOI:** 10.21769/BioProtoc.4724

**Published:** 2023-07-20

**Authors:** Laura V. Heeb, Betül Taskoparan, Antonios Katsoulas, Michal Beffinger, Pierre-Alain Clavien, Sebastian Kobold, Anurag Gupta, Johannes Vom Berg

**Affiliations:** 1 Department of Visceral Surgery and Transplantation University Hospital Zurich, Zurich, Switzerland; 2 Institute of Laboratory Animal Science, University of Zurich, Zurich, Switzerland; 3 Division of Clinical Pharmacology, Department of Medicine IV, Klinikum der Universität München, München, Germany; 4 German Cancer Consortium (DKTK), partner site Munich, Munich, Germany

**Keywords:** CRISPR/Cas9, HDR template, Mouse transgenesis, Knockout, PD-L1, Restriction site, Frameshift

## Abstract

The immune-inhibitory molecule programmed cell death ligand 1 (PD-L1) has been shown to play a role in pathologies such as autoimmunity, infections, and cancer. The expression of PD-L1 not only on cancer cells but also on non-transformed host cells is known to be associated with cancer progression. Generation of PD-L1 deficiency in the murine system enables us to specifically study the role of PD-L1 in physiological processes and diseases. One of the most versatile and easy to use site-specific gene editing tools is the CRISPR/Cas9 system, which is based on an RNA-guided nuclease system. Similar to its predecessors, the Zinc finger nucleases or transcription activator-like effector nucleases (TALENs), CRISPR/Cas9 catalyzes double-strand DNA breaks, which can result in frameshift mutations due to random nucleotide insertions or deletions via non-homologous end joining (NHEJ). Furthermore, although less frequently, CRISPR/Cas9 can lead to insertion of defined sequences due to homology-directed repair (HDR) in the presence of a suitable template. Here, we describe a protocol for the knockout of PD-L1 in the murine C57BL/6 background using CRISPR/Cas9. Targeting of exon 3 coupled with the insertion of a HindIII restriction site leads to a premature stop codon and a loss-of-function phenotype. We describe the targeting strategy as well as founder screening, genotyping, and phenotyping. In comparison to NHEJ-based strategy, the presented approach results in a defined stop codon with comparable efficiency and timelines as NHEJ, generates convenient founder screening and genotyping options, and can be swiftly adapted to other targets.

## Background

Programmed cell death ligand 1 (PD-L1), also known as CD274, is known to control adaptive immune responses during various pathological conditions such as autoimmune diseases, infections, and cancer (Francisco et al., 2010; Jubel et al., 2020). In particular, higher expression of PD-L1 on antigen-presenting cells as well as on cancer cells is known to engage with PD-1 on activated CD8+ T cells, thereby inhibiting their cancer response (Han et al., 2020). Thus, the study of the role of PD-L1 in cancer development and progression was and still is of utmost importance. For this, loss-of-function mouse mutants are invaluable tools whose generation had been time and resource intense for decades.

CRISPR/Cas9 (clustered regularly interspaced short palindromic repeats/Cas9) is an RNA-guided nuclease system that has been adapted to be a potent gene editing tool. It has quickly evolved to be the method of choice for targeted gene editing and genome-wide screens. It is superior to previously used methodologies such as homologous recombination in embryonic stem cells or use of ZNF (zinc fingers) (Lee et al., 2010; Meyer et al., 2010; Söllü et al., 2010) and TALENs (transcription activator-like effector nucleases) (Cermak et al., 2011; Zhang et al., 2011). Compared to ZNF and TALENs, it relies on DNA–RNA heteroduplex formation rather than protein–DNA interaction (Cong et al., 2013; Jinek et al., 2013; Mali et al., 2013). Here, we show a protocol using the CRISPR/Cas9 system to specifically knockout PD-L1 in the C57BL/6 mouse. After the CRISPR/Cas9-mediated dsDNA break, cell-intrinsic DNA repair mechanisms such as non-homologous end joining (NHEJ) and homology-directed repair (HDR) will take place. During NHEJ-based repair, the CRISPR/Cas9-induced double-strand break is repaired by random insertion or deletion of nucleotides at the cut site, which should in theory result in two out of three cases to a frameshift and premature translational stop. This method enables simple and fast generation of loss-of-function alleles and has been rapidly adapted to generate mouse mutants but does not generate precise gene edits. Compared to NHEJ, which leads to random heterogenous outcomes and requires sequencing of the targeted locus when screening founder animals, HDR is template based and more precise, allowing the insertion of specific sequences into the DNA break (Miyaoka et al., 2016; Yang et al., 2020). Here, we show the targeted deletion of exon 3 by insertion of a HindIII restriction site that leads to a defined stop-codon by homologous recombination. This leads to a functional knockout of PD-L1 and can be easily screened by PCR amplification of the targeted locus and a subsequent restriction digest. This protocol can be adapted to target PD-L1 in other mouse strains or cell lines and, most importantly, to target other genes of interest.

## Materials and reagents

1. Flat PCR caps, 8–250 strips (Thermo Fisher Scientific, catalog number: AB0784)
2. Qiagen DNeasy blood & tissue kit (Qiagen, catalog number: 69504)
3. Qiagen Taq PCR core kit (Qiagen, catalog number: 201225).
*Note: Alternatively, high fidelity polymerases such as Pfu (Promega, catalog number: M7741), Phusion (NEB, catalog number: M0530), or Q5 (NEB, catalog number: M0491) can be used to decrease probability of amplification errors, which may increase the accuracy of Sanger sequencing.*
4. Oligos and primers (Table 1) (iDT)
5. *Streptococcus* Pyogenes Cas9, 2× NLS SpCas9 (New England Biolabs, catalog number: M0646)
6. TE buffer, RNase free (Invitrogen, catalog number: 12090-015)
7. Agarose, LE, analytical grade (Promega, catalog number: V3125)
8. DNA dye (gel loading dye Purple 6×) (New England BioLabs, catalog number: B7024S)
9. Restriction enzyme (HindIII) (New England Biolabs, catalog number: R0104T)
10. Restriction enzyme buffer (2.1) (New England Biolabs, catalog number: R0104T)
11. DNA ladder (Mass Ruler Low Range DNA Ladder) (Thermo Fisher Scientific, catalog number: SM0311)
12. Fluorescently labeled antibody against PD-L1 (clone 10F.9G2, PE-Dazzle594 conjugate) (BioLegend, catalog number: 124324)
13. Fluorescent reagent to discriminate cell viability (Zombie Aqua) (BioLegend, catalog number: 423101)
14. Erythrocyte lysis buffer (RBC lysis buffer) (BioLegend, catalog number: 420302)
15. Recombinant murine interferon γ (IFNγ) (PeproTech, catalog number 315-05)
16. ddH_2_O
17. Microinjection buffer (see Recipes): Tris-base (Biosolve, catalog number: 20092391), HCL (Merck Millipore, catalog number: 109057), EDTA (Invitrogen, catalog number: 15575-038)
18. TAE buffer (see Recipes): Tris-base (Biosolve, catalog number: 20092391), acetic acid (Sigma-Aldrich, catalog number: 33209), EDTA (Invitrogen, catalog number: 15575-038)

## Equipment

1. Biometra PCR thermocycler (Bio-Rad, model: C1000 Touch Thermal Cycler)
2. Centrifuge (Vaudaux-eppendorf, catalog number: 5418/0005108)
3. NanoDrop (DeNoVix DS-11 + spectrophotometer)
4. Gel chamber (Bio-Rad, Sub-Cell GT)
5. Machine to run gel (BioRad Power-Pac Basic)
6. Machine to image gel (Quantum ST4, 1120 – Skylight Xpress)
7. Flow cytometer (LSR Fortessa, BD)

## Software

1. CLC Genomics Workbench (version 22, Qiagen, https:///www.qiagen.com/)
2. Ensembl (http://www.ensembl.org/index.html)
3. CRISPOR (http://crispor.tefor.net/)
  *Note: For all three points, various alternatives exist: snapgene (**https://www.snapgene.com/**) for point one; for point two, we suggest UCSC genome browser (**https://genome.ucsc.edu/**); for point three, chopchop (**http://chopchop.cbu.uib.no/*)
4. FlowJo (version 10, BD)

## Procedure

- **Design of crisprRNA targeting exon 3 of CD274 and 4-base pair HindIII restriction site insert**
  1. Search CD274 on an appropriate genome browser (e.g., www.ensembl.org or www.genome.ucsc.edu) and download gene sequence. Check the gene for transcript variants, exons/introns, and corresponding protein domains. Cd274 has one protein-coding transcript (Ensembl Gene: CD274 ENSMUSG00000016496; Ensembl Transcript: CD274-201), which has seven exons.
  2. Open CD274 sequence on CLC Genomic Workbench and annotate regions of interest [e.g., exons of targeted transcript with corresponding open reading frames (ORF)].
  3. Choose the location you want to target (i.e., exon 3) (Figure 1).
  4. Copy the sequence you want to target into an appropriate guideRNA design tool (e.g., www.crispor.tefor.net). Choose guide sequence length, the protospacer adjacent motif (PAM site), the Cas9 protein you want to use (i.e., 20bp-NGG–Sp Cas9), and the latest assembly of the respective *Mus musculus* reference genome (i.e., UCSC Dec. 2011 mm10=C57BL/6J). PAM is a short DNA sequence (2–6 bp, depending on the Cas enzyme) following the DNA region targeted for cleavage by CRISPR/Cas9 and, in case of the *Streptococcus* pyogenes (Sp) Cas9 nuclease, can be found 3–4 nucleotides downstream of the predicted cut site. While the guide sequence defines the crRNA or guideRNA–DNA interaction, it is essential for the DNA–protein interaction.
  5. Choose the best guide sequence to use (see guide sequence “guide_61/fw_ex3” in Table 1 and Figure 1).
*Note: The goal is to get a double-strand break that leads to homologous recombination, so that a ssDNA donor repair template containing a 4-base pair HindIII restriction site with 57 bp (left) and 50 bp (right) homology arms on either side can be inserted. This will lead to a +1 frameshift and a stop codon 75 bp downstream of the restriction site (Figure 2).*
    **Figure 1.**
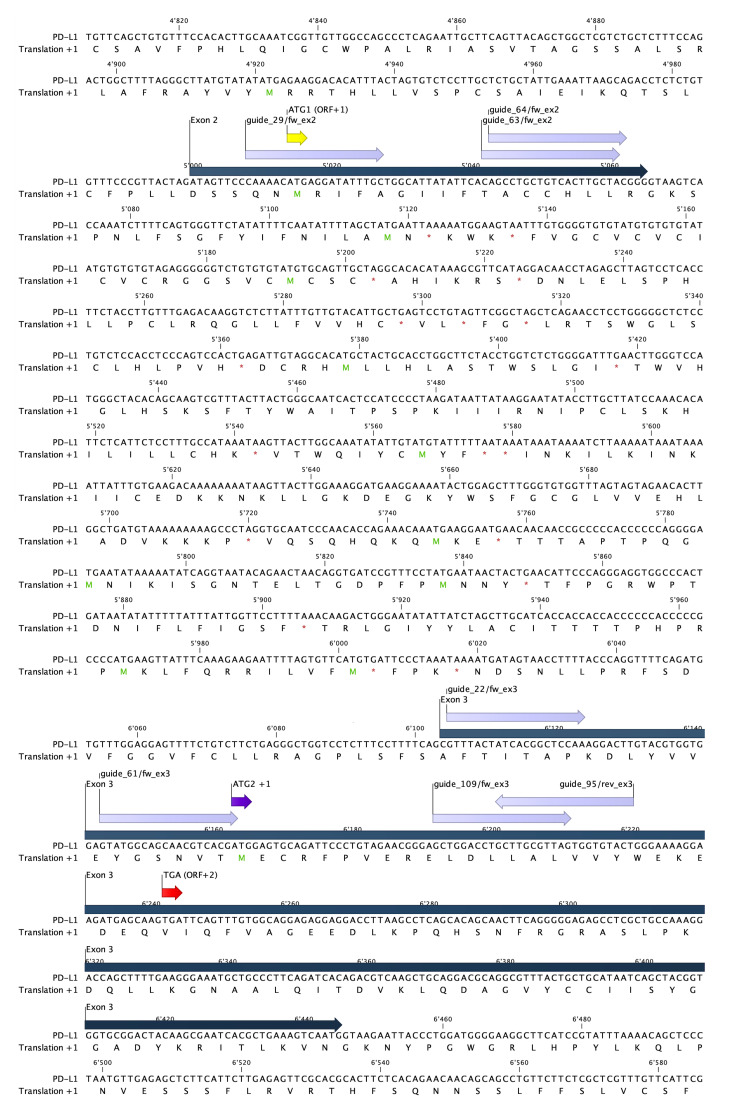
Genomic landscape of potential targets in exon 2 and 3 of wildtype PD-L1 with possible guide sequences. Sequence of exon 2 and 3 from CD274 with corresponding open reading frames (ORF) (+1) as depicted in CLC Genomics Workbench (dark blue). Exons are depicted in blue and start codons for ORF+1 are depicted in yellow and purple (ATG1 and ATG2, respectively). Possible guide sequences for targeting crRNA are depicted in lilac. Alternative stop codon (TGA ORF+2), 72 bp downstream of guide_61/fw_ex3-induced cutting site (in between A and C, on position 57 and 58 of exon 3), is depicted in red.
    **Table 1.** Oligos and primers

Name	Application	Sequence (5′–3′)	Length (bp)	Manufacturer
guide_61/fw_ex3	crRNA	GTATGGCAGCAACGTCACGA	20	Alt-R CRISPR, iDT
tracrRNA	tracrRNA	Sequence according to manufacturer	67	Alt-R CRISPR, iDT
HDR template HindIII +1fs	ssDNA homology-directed repair donor template	CGTTTACTATCACGGCTCCAAAGGACTTGTACGTGGTGGAGTATGGCAGCAACGTCAAGCTTGGAGTGCAGATTCCCTGTAGAACGGGAGCTGGACCTGCTTGCGTTAGTG	111	Megamer, iDT
guideRNA_61/fw_Left	Fw Primer	CCCCGCCCCATGAAGTTATT	20	Microsynth
guideRNA_61/fw_Right	Rv Primer	TGCAGCTTGACGTCTGTGAT	20	Microsynth
    **Figure 2.**
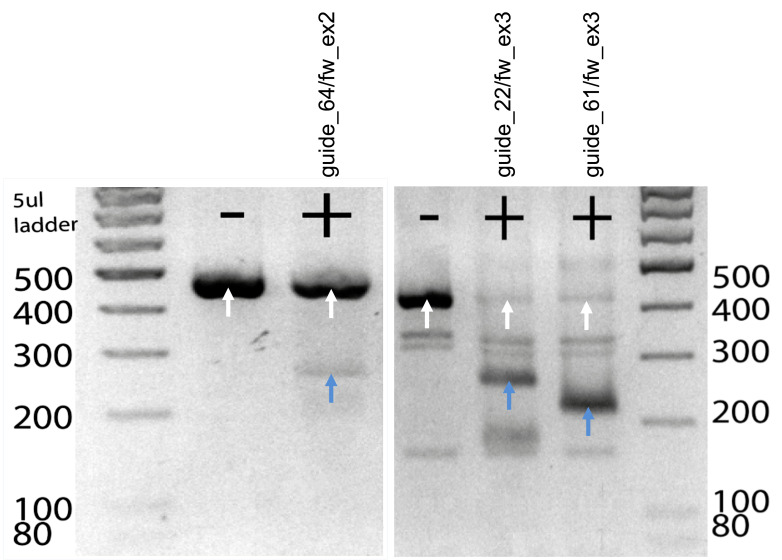
In vitro digestion of candidate guideRNAs to test cutting efficiency. Target DNA was digested for 20 min at 37 °C with a cocktail consisting of crRNA, tracrRNA, and Cas9 enzyme. Digestion products were run on a gel and cutting efficiency was assessed based on the resulting bands. Uncut amplicons (white arrows) show a band at 400 bp and the digested products (blue arrows) show a band at 200 bp.
  6. Annotate chosen guide sequence in CLC Genomics (Figure 1).
  7. Annotate ssDNA donor repair template. See sequence “HDR template HindIII +1fs” in Table 1 and Section C.
  8. Order lyophilized crRNA and the universal tracrRNA. Note that tracrRNA does not confer target specificity, so it can be ordered in bulk and combined with different, target-specific crRNAs.
- **Test guideRNA by in vitro digest**
  1. Extract DNA from murine cells or biopsy with an appropriate DNA extraction kit (e.g., Qiagen DNeasy blood & tissue kit).
  2. Amplify DNA target site from extracted DNA template with the Qiagen Taq PCR core kit and appropriate forward and reverse primers (Table 1). Location and sequence of primers and expected 412 bp amplicon is shown in Figure 1. See PCR reagents in Table 2 and conditions in Table 3.
    **Table 2.** Concentrations and volumes for target DNA PCR amplification

Reagent	Concentration	Volume (25 μL total/reaction)
guideRna168forwLe	10 μM	1.25
guideRNA168forwRi	10 μM	1.25
dNTPs	200 μM	0.5
10x buffer		2.5
Taq polymerase	2.5 U	0.125
ddH_2_O		18.375
DNA		1
    **Table 3.** PCR Conditions

Step	Time (s)	Temperature (°C)
1	60	98
2	10	98
3	30	65
4	30	72
Go to step 2 35×		
5	120	72
6	Up until use	4
  3. Dilute lyophilized crRNA and tracrRNA (usually 2 nmol) to 100 μM stock concentration in TE buffer. Then, further dilute to 10 μM working concentration in TE buffer.
*Note: Working with RNA requires RNase-free working conditions, as RNA is not very stable and can be degraded by RNases very quickly. RNA samples should always be stored on ice and only diluted in RNase-free reagents. The crRNA and tracrRNA can be diluted and aliquoted in TE buffer and stored at -80 °C for up to a year.*
  4. Per reaction, in a PCR reaction tube, incubate 3.68 μL of crRNA with 1.84 μL of tracrRNA in 5 μL of microinjection buffer at 78 °C for 10 min on a PCR heating block, followed by 37 °C for 30 min (Mix 1).
*Note: For uncut control, include one reaction to which you do not add crRNA. Replace this volume (3.68 μL) with ddH_2_O.*
  5. Remove from heating block and slowly cool down to room temperature (RT) for 15 min.
  6. Take 10.7 μL of Mix 1 and add 0.5 μL of Cas9 (20 μM) and 38.8 μL of ddH_2_O. Pipette up and down a couple of times and incubate for 10 min at RT (Mix 2).
  7. Take 15 μL of Mix 2 and add 2 μL of Cas9 10× buffer and 400–500 ng of target DNA and fill up with ddH_2_O to 20 μL.
*Note: With a final volume of 20 μL, maximum 3 μL of DNA target and 15 μL of Mix 2 can be added. When the concentration of the target DNA is low and e.g., 6 μL needs to be added to reach 400–500 ng, increase the Cas9 10× buffer to e.g., 3 μL, Mix 2 to 22.5 μL, and final volume to 30 μL.*
  8. Incubate at 37 °C for 60 min and load onto 2% agarose gel (2 g of agarose/mL of TAE buffer).
  9. Add loading dye to the samples and load DNA ladder and samples onto the gel. Run the gel at 100 V until bands have separated well.
*Note: The in vitro digestion allows you to evaluate the potential of your guide for in vivo cutting efficiency. You get an idea of the cutting efficiency by comparing the intensity of the uncut amplicon to the two cutting products. When a guide already does not cut the target amplicon in an in vitro digest, the probability of efficient cutting in vivo might also be low. Likewise, when comparing two guides for the same DNA target site, a combination of specificity score and cutting efficiency will help to choose the optimal guide. Expected band sizes are approximately 400 bp for the uncut amplicon and 200 bp for the cut product (Figure 2).*
- **Design ssDNA oligo as HDR donor template to integrate defined frameshift and enzyme restriction site**
  1. Choose appropriate guide sequence to target locus of interest.
*Note: For a complete loss-of-function phenotype, a good guide sequence does not target a locus in front of an alternative start site (ATG), a locus including SNPs, or a locus close to the C-terminal site. Furthermore, it is important to not target non-constitutive exons (exons not present in all isoforms of the gene) (Doench, 2018). We targeted exon 3, as these guide sequences generally show higher specificity and efficacy than guides targeting exon 2. We chose guide_61/fw_ex3, as this crRNA has the highest specificity and efficacy score and less off targets than guide_109/fw_ex3 depicted by CRISPOR (Table 4).*
    **Table 4.** Potential crRNAs with respective target loci, sequence and specificity, and efficacy scores according to CRISPOR. The most promising crRNAs (*) have been tested by in vitro digestion (Figure 2). Two asterisks (**) mark the crRNA chosen for zygote microinjection.

crRNA	Target	Sequence	MIT	CFD	Doench ‘16	Mor.-Mateos
guide_29/fw_ex2	Exon 2	CAAAACATGAGGATATTTGC	68	79	44	12
guide_63/fw_ex2	Exon 2	CAGCCTGCTGTCACTTGCTA	68	82	46	56
guide_64/fw_ex2*	Exon 2	AGCCTGCTGTCACTTGCTAC	79	91	48	36
guide_22/fw_ex3*	Exon 3	GTTTACTATCACGGCTCCAA	91	96	63	41
guide_61/fw_ex3**	Exon 3	GTATGGCAGCAACGTCACGA	96	96	66	44
guide_109/fw_ex3	Exon 3	GCTGGACCTGCTTGCGTTAG	94	97	42	66
guide_95/rev_ex3	Exon 3	AGTACACCACTAACGCAAGC	93	97	60	9
  2. Design ssDNA donor repair template:
    - To induce homology-directed repair and generate a +1 frameshift, design a ssDNA template that spans the targeted locus and cutting site with two homologous arms of 57 bp (left) and 50 bp (right).
*Note: The double-strand break will be between position 17 and 18 of the protospacer (guide) sequence. This might differ according to the type of Cas9 and guideRNA used. We used Sp Cas9 that has a cutting site between the third and the fourth bp upstream of the PAM site of guide_61/fw_ex3 (TGG).*
    - Add an insert of 1 or 4 bp in between the two homology arms to induce a frameshift (see “HDR template HindIII +1fs” in Table 1 and Figure 3).
*Note: We designed the insert and homology arms in a way that 3 bp of the wildtype sequence (CGA) get replaced by a 4 bp HindIII restriction site (AGCT), thereby conveniently replacing the A of the start codon in exon 3 with the T of the restriction site.*
    - Add homology arms with 57 bp (left) and 50 bp (right) homologous sequences.
  3. Order ssDNA donor template.
    **Figure 3.**
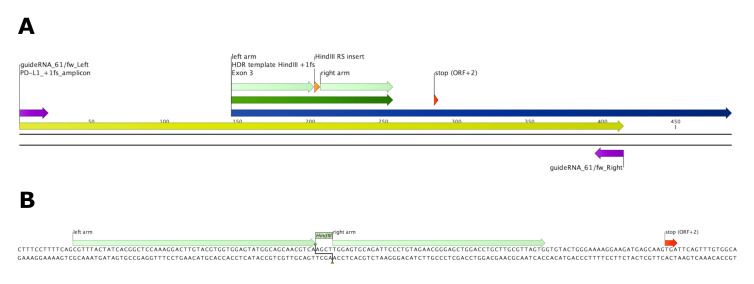
Induction of +1 frameshift and premature translational stop by homology-directed repair (HDR) of ssDNA template. (A) Overview of exon 3 (dark blue) and 414 bp amplicon (yellow) spanning the homology repair-directed template insert (HDR template HindIII +1fs, dark green, homology arms depicted in light green) carrying a HindIII restriction site (orange). Alternative stop codon [stop (ORF +2)] on position 138–141 of wildtype PD-L1 exon 3 is depicted in red. Primers spanning the amplicon are depicted in purple. (B) Sequence of 111 bp HDR template Hind III +1fs as depicted in CLC Genomics Workbench. 57 bp (left) and 50 bp (right) homology arms span the 4 bp (AGCT) HindIII restriction site insert. Insertion of 4 bp HindIII restriction site replacing 3 bp (CGA) of wildtype PD-L1 leads to a +1 frameshift and a premature stop of translation due to the alternative stop codon in ORF +2.
- **Preparation of microinjection mix with CRISPR/Cas9 ribonucleoprotein particle (RNP) plus HDR oligo repair template**
  1. Resuspend lyophilized crRNA and tracrRNA in 1× microinjection buffer to a final concentration of 10 μM:
    - Mix a total of 1.84 μL of crRNA and tracrRNA with 5 μL of 10× injection buffer and 0.5 μL of *Streptococcus* pyogenes Cas9 protein (20 μM) and subsequently incubate for 15 min at 37 °C.
    - For knock-in mouse production, add 500 ng of ssDNA donor template (HDR template HindIII +1fs) after incubation.
    - Dilute the mix with ddH_2_O to a final volume of 50 μL.
  2. Spin the final mix down at 21,000× *g* for 3 min at RT.
  3. Keep injection mix at RT during the injection procedure.
*Notes:*
    - *The microinjection procedure was performed at the transgenesis core of the University of Zürich, Switzerland and cannot be performed with simple laboratory equipment. A short description of the procedure is found in section E. For a more detailed description of the reproductive biology and the microinjection procedure, please refer to the transgenesis core of your research facility.*
    - *As a quality control measure after the microinjection procedure, the enzymatic activity of the injection mix (can be stored at -80 °C) can be tested by adding target DNA and essentially performing an in vitro digestion as described in section B.*
- **Zygote microinjection, embryo culture, and retransfer into pseudopregnant foster animals**
  Microinjection was performed at the transgenesis core of the University of Zürich, Institute of Laboratory Animal Science under license of the cantonal veterinary office in accordance with federal law. C57BL/6 mice at 3–4 weeks of age (Charles River Laboratories, Germany) were super ovulated by intraperitoneal injection of 5 IU of pregnant mare serum gonadotropin (Folligon, MSD Animal Health GmbH, Luzern, Switzerland) followed 48 h later by injection of 5 IU of human chorionic gonadotropin (Pregnyl, MSD Animal Health GmbH, Luzern, Switzerland). Mouse zygotes were obtained by mating C57BL/6J stud males with superovulated C57BL/6 females. Zygote microinjections (pronuclear injection into the male pronucleus), embryo culture o/n, and retransfer of 2-cell stage embryos into pseudopregnant foster animals via surgical embryo transfer were performed according to standard mouse transgenesis protocols (e.g., Harms et al., 2014; Quadros et al., 2018).
- **Screening of founders by PCR followed by HindIII digest of the amplicon**
  1. Take biopsies of the resulting founder pups after zygote implantation.
  2. Amplify the DNA target site by PCR with Qiagen Taq PCR core kit (Table 2 and Table 3). This leads to a 414 (mutated) or 413 (wild type) bp amplicon.
*Note: As a negative control, DNA template in PCR mix can be substituted with 1 μL of ddH_2_O.*
  3. After amplification, carefully open the PCR strip and add 0.5 μL of HindIII restriction enzyme/reaction, close reaction tubes again, and incubate at 37 °C in the PCR machine overnight. Cave: confirm on the NEB table (https://international.neb.com/tools-and-resources/usage-guidelines/activity-of-restriction-enzymes-in-pcr-buffers) the activity of HindIII in your Taq buffer. Depending on your Taq, you might need to add HindIII reaction buffer to ensure proper activity.
  4. Prepare 1.5% agar gel (1.5 g of agarose/mL TAE buffer).
  5. Load DNA ladder and samples with added loading dye onto the gel.
  6. Run at 100 V until bands of standard size have separated well.
*Note: In vitro digestion of amplicon with inserted HindIII restriction site leads to cutting of the 414 bp amplicon into approximately 200 bp fragments. If the donor template has integrated successfully, we expect one band at 200 bp, while the unintegrated wildtype band stays at 413 bp due to the missing HindIII restriction site. Therefore, amplicons derived from wildtype mice will be visible on the gel as one band at 413 bp. Amplicons derived from homozygous mutants will show one band at 200 bp, while heterozygous mutants will show both bands at 200 bp and 413 bp, respectively (Figure 4).*
    **Figure 4.**
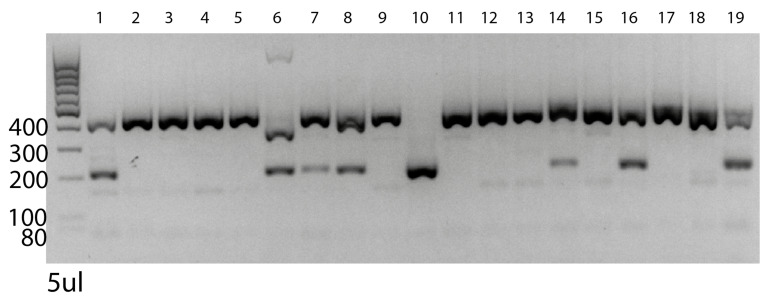
PCR validation of PD-L1 knockout founder pups. Target gene was amplified and subsequently digested with HindIII restriction enzyme. Numbers 1–19 represent the different founder pups. The left side shows DNA ladder with depicted sizes. 400 bp bands show wildtype alleles, while 200 bp bands represent cut alleles that integrated the ssDNA donor template with HindIII restriction site. Subsequent digestion with HindIII restriction enzyme after PCR cuts the alleles harboring the de novo integrated HindIII 200 bp fragments. Heterozygous pups show two bands; homozygous pups show one band. Only pup number 10 has both mutated alleles.
- **Confirm frameshift via Sanger sequencing**
  1. Prepare your samples for sanger sequencing:
    - Isolate DNA from founder pups and amplify it to get amplicon as described in step F2.
*Note: Sanger sequencing methods are most precise when DNA is approximately 300–1,000 bp. Choose your primers accordingly.*
    - Check quality of DNA template by UV absorption using a NanoDrop.
*Note: Good-quality DNA will have an A_260_/A_280_ ratio of 1.7–2.0.*
    - Dilute the DNA template to desired concentration according to the protocol of your sequencing provider.
*Note: A general rule is 1.5 ng/μL per 100 bp. As our amplicon is 414 bp long, 6–7.5 ng/μL should suffice.*
    - Dilute your primers according to the protocol of your sequencing provider.
*Note: The general rule is 4 μM for premixed primers and 10 μM for separate primers.*
    - Send the DNA amplicons and primers in for sequencing.
  2. Import sequencing files into CLC Genomics Workbench.
  3. Align sequencing files with the sequence of the amplicon and check for conflicts (Figure 5).
    **Figure 5.**
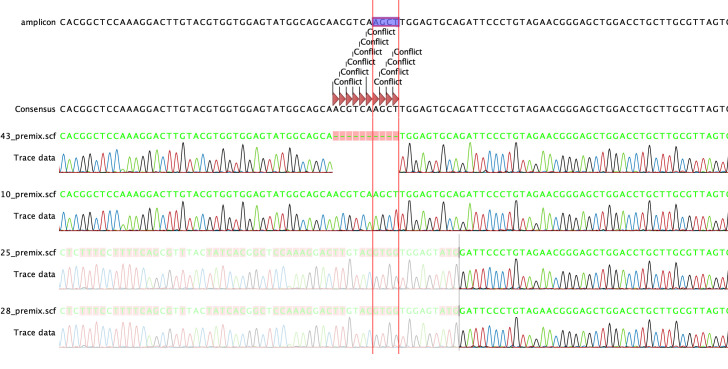
Confirmation of +1 frameshift in founder pups via Sanger Sequencing. The alignment of the sequencing data to the amplicon shows a clean incorporation of the insertion template with the HindIII restriction site AGCT into the genome of founder pup number 10 (10_premix.scf).
- **Backcross founder pups with clean insertions to C57BL/6 mice**
  1. Backcross founders once to wild type to minimize potential off-target effects:
    - Mate positive founders with C57BL/6 wildtype mice.
    - Perform routine genotyping as described in section F and choose heterozygous mice for subsequent het × het crossing to yield homozygous mutants (Figure 6).
  **Figure 6.**
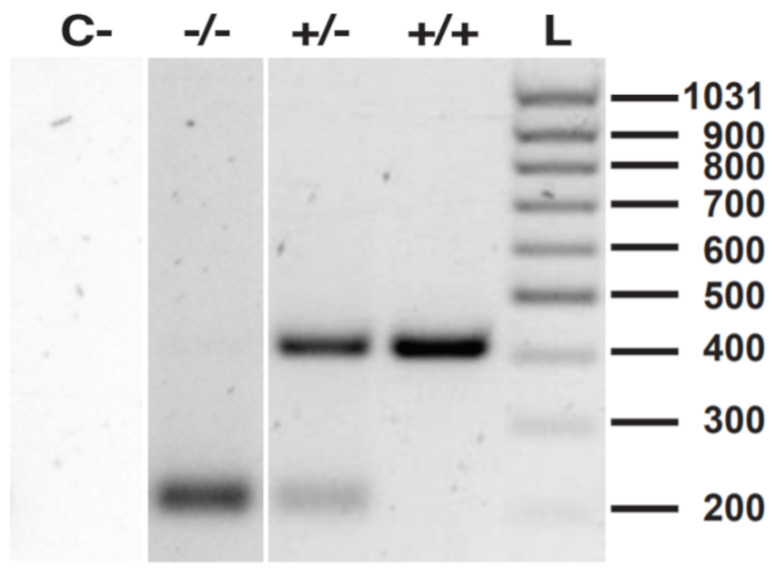
Routine genotyping by PCR and HindIII digestion. C- shows negative control (PCR and HindIII restriction reaction without DNA template); -/- shows homozygous mutant with 200 bp fragments; +/- shows heterozygous mutant with both 200 bp and 400 bp fragments; +/+ shows wildtype gene with 400 bp fragments; L is the DNA ladder.
- **Optional: confirm phenotypic knockout in homozygous PD-L1 mutants by flow cytometry**
  1. Collect blood.
  2. Perform lysis of erythrocytes using RBC lysis buffer according to manufacturer’s instructions.
  3. Resuspend cells in medium containing 10 ng/mL murine IFNγ.
*Note: IFNγ stimulation leads to a strong upregulation of PD-L1 on the surface of cells.*
  4. Stain the cells with Zombie Aqua and primary fluorophore-conjugated antibodies.
*Note: In general, it is enough to only stain the cells with a viability dye and an anti-PD-L1 antibody. However, it is recommended to also stain the cells for immune cell markers such as CD45, CD3, and CD11b to discriminate between populations. General protocols for surface staining can be found on the websites of flow cytometry antibody providers.*
  5. Acquire cells on flow cytometer.
  6. Gate on cells of interest in FlowJo (Figure 7A).
  7. Check PD-L1 expression (Figure 7B).
    **Figure 7.**
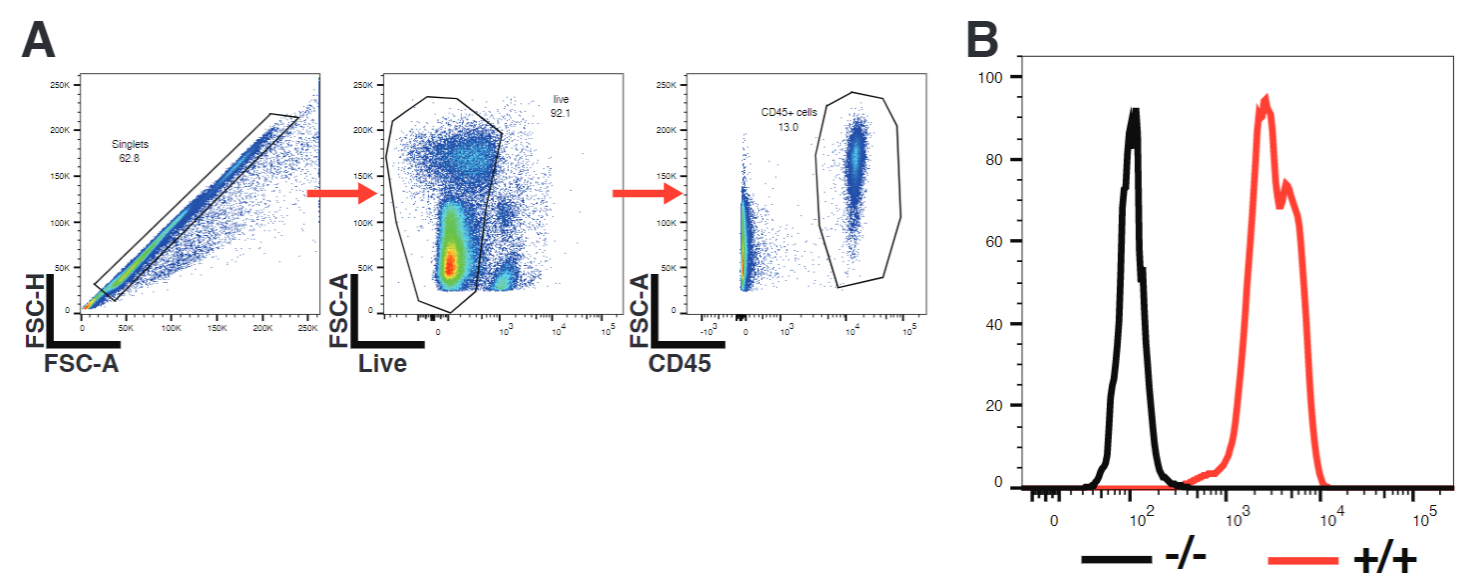
Confirmation of phenotypic knockout of PD-L1. Mouse peripheral blood mononuclear cells (PBMCs) were stimulated overnight with 10 ng/mL IFNγ and consequently surface-stained for PD-L1. (A) Gating strategy. (B) Representative overlay of histograms of PD-L1 expression of CD45+ immune cells as gated in A), showing PD-L1-/- cells in black (-/-) and wildtype cells in red (+/+).

## Notes

The described procedure can be adapted in various ways. For example, instead of introducing a restriction enzyme recognition site, an already existing restriction enzyme recognition site in the open reading frame of interest may also be destroyed by an indel by simply leaving away the HDR template. Such a simple non-homology directed repair event may lead to a frameshift, resulting in a loss of protein expression as well. However, screening for loss of restriction sites does not guarantee a particular frameshift and can even lead to in-frame insertions or deletions. The method described here circumvents this problem by introducing a novel restriction site together with a particular frameshift. This allows for convenient screening for these defined, desired frameshifts at minimal trade off with regards to targeting efficiency, since the insert is very small.

Most importantly, this method can be adapted to other genes of interest, targeted insertion of larger genomic regions, as well as to generation of defined mutants in cell lines, where targeting both alleles is more crucial than for mouse transgenesis.

## Recipes

**Buffers and solutions**

1. **1× microinjection buffer**
  10 mM tris-HCl (pH 7.5) and 0.1 mM EDTA. Tris-HCL and EDTA can be used again up to six months after preparation; the microinjection buffer should be mixed fresh every time.
2. **50× TAE (Tris-acetate-EDTA) buffer**

Dissolve 242 g of Tris base in 700 mL of ddH_2_O. Add 57.1 mL of 100% acetic acid and 100 mL of 0.5 M EDTA (pH 8.0). The pH should be approximately 8.5; if not, adjust pH. Adjust the solution to a final volume of 1 L. After preparation, solution can be stored and used at RT for up to six months.
